# Comprehensive Genetic Study of Malignant Cervical Paraganglioma

**DOI:** 10.3390/ijms24098220

**Published:** 2023-05-04

**Authors:** Anastasiya Snezhkina, Vladislav Pavlov, Maria Fedorova, Dmitry Kalinin, Elena Pudova, Anastasiya Kobelyatskaya, Ildar Bakhtogarimov, George Krasnov, Anna Kudryavtseva

**Affiliations:** 1 Engelhardt Institute of Molecular Biology, Russian Academy of Sciences, 119991 Moscow, Russia; vladislav1pavlov@gmail.com (V.P.); fedorowams@yandex.ru (M.F.); pudova_elena@inbox.ru (E.P.); kaa.chel@mail.ru (A.K.); bakhtogarimov@gmail.com (I.B.); gskrasnov@mail.ru (G.K.); rhizamoeba@mail.ru (A.K.); 2 Vishnevsky Institute of Surgery, Ministry of Health of the Russian Federation, 117997 Moscow, Russia; dmitry.v.kalinin@gmail.com

**Keywords:** head and neck paraganglioma, middle ear paraganglioma, jugulotympanic paraganglioma, malignant paraganglioma, succinate dehydrogenase, *SDHx*, *SDHB*, LOH, mutational load

## Abstract

Malignant middle ear paraganglioma (MEPGL) is an exceedingly rare tumor of the neuroendocrine system. In general, MEPGLs represent as slow growing and hypervascularized benign neoplasms. The genetic basis of MEPGL tumorigenesis has been poorly investigated. We report a case of malignant MEPGL accompanied by the comprehensive genetic analysis of the primary tumor and metastasis. Based on whole-exome sequencing data, the germline pathogenic mutation p.R230H in the *SDHB* gene, encoding for subunit B of mitochondrial complex II, was found in a patient. Analysis of somatic mutation spectra revealed five novel variants in different genes, including a potentially deleterious variant in *UNC13C* that was common for the tumor and metastasis. Identified somatic variants clustered into SBS1 and SBS5 mutational signatures. Of note, the primary tumor was characterized by Ki-67 4% and had an elevated mutational load (1.4/Mb); the metastasis’ mutational load was about 4.5 times higher (6.4/Mb). In addition, we revealed somatic loss of the wild-type *SDHB* allele, as well as loss of heterozygosity (LOH) at the 11p locus. Thus, germline mutation in *SDHB* combined with somatic LOH seem to be drivers that lead to the tumor’s initiation and progression. Other somatic changes identified can be additional disease-causing factors. Obtained results expand our understanding of molecular genetic mechanisms associated with the development of this rare tumor.

## 1. Introduction

Middle ear paraganglioma (MEPGL) arises from the extra-adrenal neural crest-derived paraganglia located in the middle ear or temporal bone region and is also termed jugulotympanic paraganglioma (JTPGL) [1]. MEPGLs account for approximately one third of all paragangliomas of the head and neck (HNPGLs) [2]. A small subset of cases (2%) are diagnosed with malignant MEPGLs, and up to 40% of patients with a familial history have multiple tumors (with carotid body and/or vagal paragangliomas) [3]. Malignant paraganglioma is defined only by the presence of metastases in non-neuroendocrine tissue. In HNPGLs, metastasis to distant organs occurs twice less than metastasis to lymph nodes, and it is associated with lower 5-year relative survival rate (11.8% for patients with distant metastasis vs. 76.8% for those with regional metastasis) [4]. It should be noted that all paragangliomas have malignant potential. To date, molecular genetic mechanisms underlying progression of paragangliomas remain unknown and effective metastasis biomarkers have not been revealed.

MEPGLs have been predominantly studied as a part of HNPGLs, and sparse genetic data were reported for these tumors. Metastatic dissemination in MEPGLs is a very rare event and has not been well investigated. This study reports an exceedingly rare case of malignant MEPGL supported with complex genetic analysis of the primary tumor and metastasis.

## 2. Case Presentation

### Clinical Characteristics of the Patient

A 17-year-old male first observed difficulty swallowing and speaking, as well as deviation to the left in the tongue in November 2003. Magnetic resonance imaging (MRI) revealed a mass of the left jugular foramen, size 49 × 31 × 28 mm, with probable additional component (20 × 12 × 10 mm) extending intracranially. The surgery was performed at the N. N. Burdenko National Medical Research Center of Neurosurgery of the Ministry of Health of the Russian Federation in August 2004. The major tumor mass, size of 50 × 40 × 30 mm, was excised before entering the jugular foramen. The upper pole of the tumor, grown through the jugular foramen, was impossible to mobilize, though an additional tumor node (d = 15 mm) was removed. The hypoglossal nerve was transected during the surgery. Pathomorphological study confirmed paraganglioma of the jugular foramen, with no metastasis in regional lymph nodes. In November 2004, MRI showed persistence of a neoplasm, size 24 × 20 × 14 mm, which could derive from the tumor remnant or local recurrence. Postoperatively, 30 sessions of remote radiation therapy with a total focal dose of 59 Gy were conducted in 2004, and 29 more sessions with a total focal dose of 58 Gy were conducted in 2006. The patient was dynamically observed with no signs of progression. In 2017, MRI was performed concerning back pain, and focal changes of secondary origin ranging in size from 5 to 22 mm were detected in the bodies of Th12-S3 vertebrae. According to the patient, no further examination was recommended. At the age of 32 years (spring 2019), the patient first noted panic attack-like symptoms: emotional excitability, increased heart rate, dizziness, noise, and pulsation in the left ear. In June 2019, MRI showed paraganglioma of the primary localization extending from the adjacent sections of the mastoid process of the left temporal bone to the level of the C1 vertebra, measuring 31 × 25 × 37 mm. In July 2019, positron emission tomography/computed tomography (PET/CT) with ^68^Ga-DOTATATE determined multiple lesions in the following regions: (1) liver: two large TATE-positive lesions in segments S3 and S6, 47 × 31 × 39 and 58 × 41 × 54 mm in size with standardized uptake values (SUVs) 34 and 27.6, respectively; (2) pancreas: two TATE-positive lesions in the body and tail sized 12 × 8 × 10 and 20 × 23 × 24 mm with SUVs 10.7 and 27.6, respectively, and (3) multiple pathological foci of the skeletal system with the presence of mixed restructuring located in the occipital bone, the left half of the sphenoid bone, in the vertebrae of the scanning area, the sacrum, and the right ilium (the largest SUVs were 41.9 in the body of Th4 and 46.08 in the left ilium).

**Figure 1:**
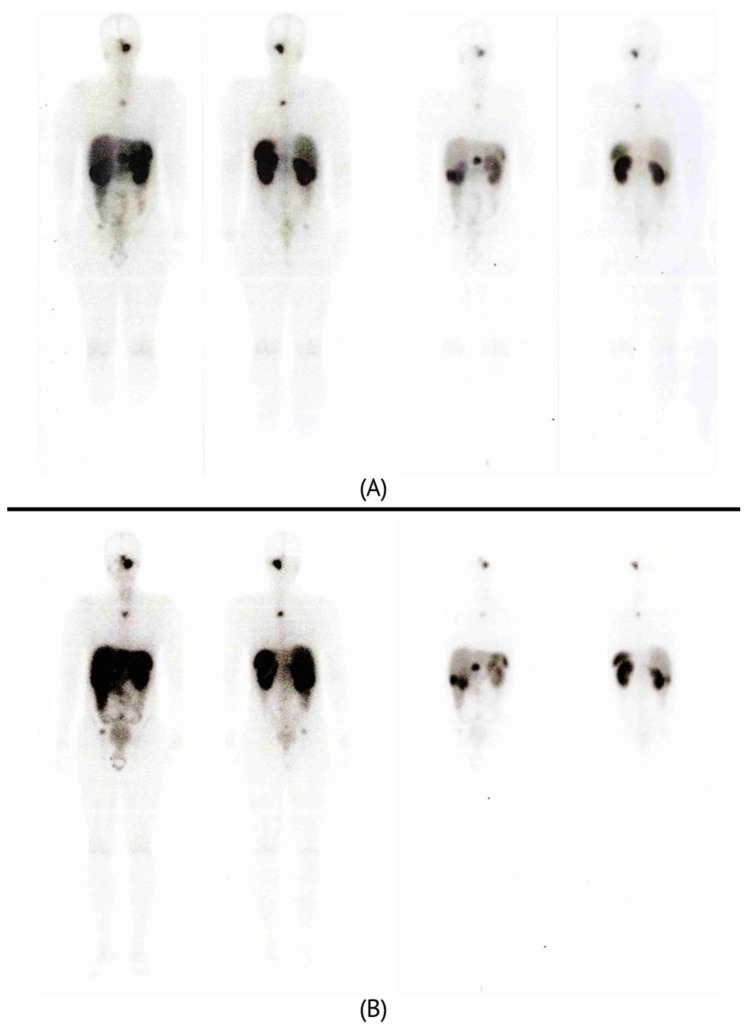
^177^Lu-DOTATATE PET/CT images, displaying the accumulation of radiopharmaceutical in neoplasms. Image (**A**) was taken during first session (September 2020) and image (**B**) was taken during fourth and last session of the therapy (March 2021). The scans on the right side represent a higher intensity of the contrast signal.

Therapy was resumed first with octreotide. In November 2019, after confirming the high-intensity accumulation of DOTATATE in most of the neoplasms, four courses of treatment with a long-acting somatostatin analogue labeled with the radionuclide ^177^Lu (^177^Lu-DOTATATE) were completed (Figure 1), which was continued with somatostatin and zoledronic acid. Subsequent PET/CT study showed stabilization of the process in terms of independent parameters, such as neoplasm size and metabolic tumor volume. Follow-up PET/CT studies were carried out each six months and up to date. The patient is currently in remission.

## 3. Results

### 3.1. Immunohistochemical Analysis of Tumor and Metastasis

**Figure 2:**
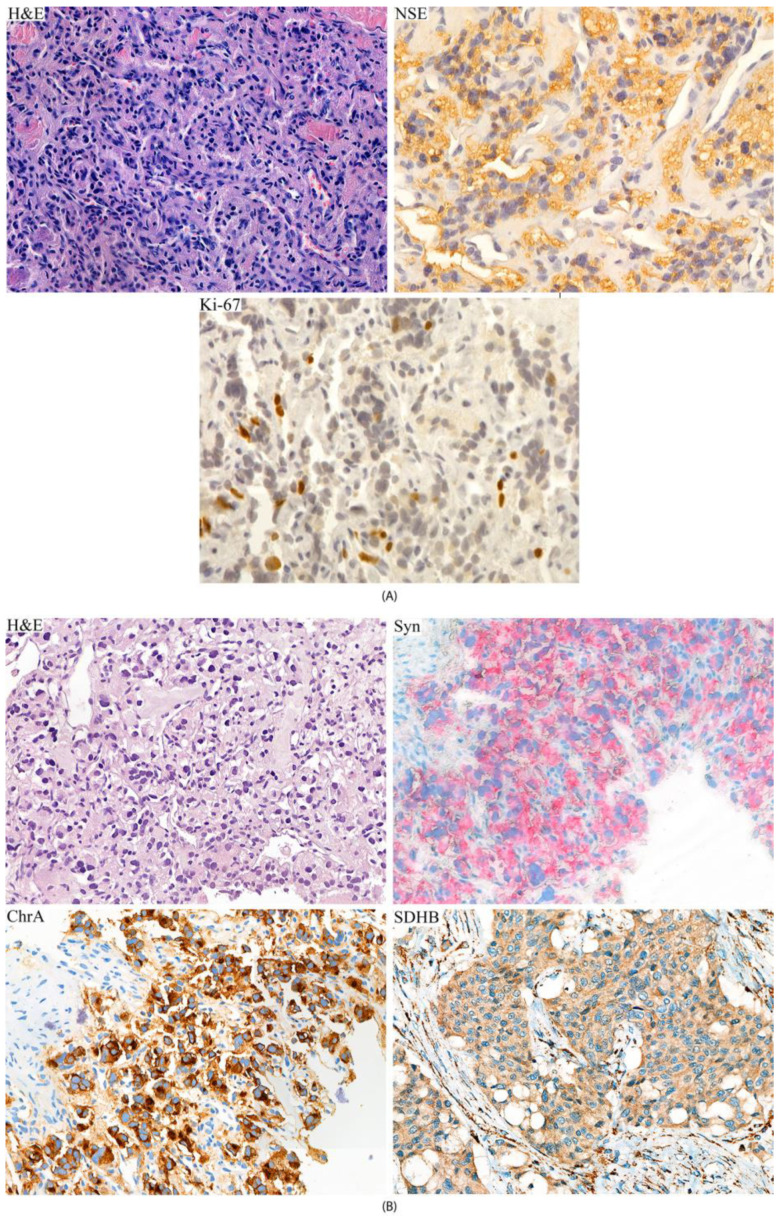
Histologic and immunohistochemical staining of primary tumor (**A**) and metastasis (**B**) of patient. (**A**) H&E, hematoxylin-eosin staining; NSE, positive IHC staining of neuron-specific enolase; Ki-67 staining of about 4% of nuclei. (**B**) H&E, hematoxylin-eosin staining; Syn, diffuse IHC staining of synaptophysin; ChrA, diffuse IHC staining of chromogranin A; *SDHB*, positive IHC staining of succinate dehydrogenase subunit B. Original magnification: 40×.

According to the pathomorphological study based on hematoxylin-eosin staining, the resected primary tumor was characterized by the nest-trabecular structure of polymorphic cells with pronounced nuclear polymorphism and anisonucleosis with developed eosinophilic granular cytoplasm (Figure 2). Mitotic activity was low; foci of necrosis were not visualized. The presence of a developed vascular network and a field of dense sclerosis/hyalinosis were noted. Immunohistochemical (ICH) staining was positive for neuron-specific enolase (NSE) [monoclonal, 22C9] (Leica Biosystems, Richmond, IL, USA), indicating a neuroendocrine tumor. The Ki-67 proliferation index [monoclonal, GM0010] (Lacopa, Russia) was about 4%.

A biopsy of a liver neoplasm was performed in August 2019. The biopsy sample was morphologically comparable to the primary tumor. Diffuse cytoplasmic expression of Synaptophysin [monoclonal, 27G12] and Chromogranin A [monoclonal, LK2H10] (GeneTex, Irvine, CA, USA) confirmed neuroendocrine origin of the biopsy tissue; more pronounced cellular nuclear polymorphism was observed (Figure 2). The metastasis sample was also analyzed for the expression pattern of *SDHB* subunits, which is a surrogate marker of *SDHx* mutations, according to a previously described procedure [5]. Immunoreaction was performed using primary monoclonal antibody [21A11AE7] from Abcam (Cambridge, MA, USA). Positive granular staining of *SDHB* was detected in the tested sample. Of note, the primary tumor sample was unavailable for examination of *SDHB* expression.

### 3.2. Mutation Profiling and Mutational Signatures

To uncover genetic variants contributing to the tumor’s development, we performed whole-exome sequencing of the primary tumor, metastasis, and blood samples from this patient. DNA from the primary tumor and metastasis was extracted using a High Pure FFPET DNA Isolation Kit (Roche, Basel, Switzerland). DNA extraction from whole blood was carried out with a MagNA Pure Compact Nucleic Acid Isolation Kit I (Roche) on a MagNA Pure Compact Instrument (Roche). Exome libraries were prepared using a TruSeq Exome Library Prep Kit (Illumina, San Diego, CA, USA) according to the manufacturer’s instructions. High-throughput sequencing was performed on an Illumina NextSeq 500 System under paired-end mode of 76 × 2 bp with a minimum coverage of 300×. Bioinformatics analysis was carried out as previously described [6] and included germline and somatic variant calling using GATK HaplotypeCaller [7] and Mutect2 [8], as well as mutational signature analysis with SigProfiler [9]. 

Twenty-four paragangliomas and pheochromocytomas (PPGLs) susceptibility genes (*EGLN1*, *EGLN2*, *MDH2*, *FH*, *SDHA*, *SDHB*, *SDHC*, *SDHD*, *SDHAF2*, *MAX*, *RET*, *TMEM127*, *VHL*, *EPAS1*, *NF1*, *H3F3A*, *IDH1*, *IDH2*, *ATRX*, *HRAS*, *SDHAF3*, *SDHAF4*, *CSDE1*, and *SLC25A11*) were analyzed for the presence of pathogenic/likely pathogenic germline variants. As a result, a heterozygous missense variant in the *SDHB* gene, NM_003000: c.G689A, p.R230H (chr1: 17349179, rs587782604), was revealed in the studied patient. The variant allele frequency (VAF) was 45% in blood sample, as well as 66% and 65% in tumor and metastasis samples, respectively. The *SDHB*: p.R230H mutation results in nonsynonymous amino acid change in the encoded protein and was predicted as damaging by all used in silico tools (Polyphen2, SIFT, LRT, MutationTaster, PROVEAN, FATHMM, DANN, CADD, and others). Frequency of the variant in the control population (gnomAD) was close to 0. This variant was previously reported in the ClinVar database as a pathogenic/likely pathogenic mutation associated with hereditary syndromes, including paragangliomas and pheochromocytomas. According to the Varsome interpretation, which is based on criteria of the American College of Medical Genetics and Genomics and the Association for Molecular Pathology (ACMG-AMP) [10,11], this variant is classified as pathogenic. The identified *SDHB* variant was validated using Sanger sequencing (Figure S1). No pathogenic/likely pathogenic variants were identified in other tested susceptibility genes.

Somatic mutation analysis was carried out for both tumor and metastasis of the patient. A total of 434 somatic variants with alternative read coverage ≥10 were identified in tumor, whereas 720 somatic variants were found in metastasis. Mutational load in tumor and metastasis, which was calculated as a weighted mutational load (wML) according to the previously described algorithm [12], was 1.4 and 6.4 mutations per megabase (Mb) at VAF 15%, respectively (under threshold of VAF 20%, wML was 0.5/Mb for primary tumor and 2.8/Mb for metastasis). 

We identified five novel somatic potentially deleterious variants that passed the following filters: population frequency ≤1%, pathogenicity score ≥0 (summary score from pathogenicity prediction tools, position conservation score, and clinical significance), exonic/splicing/frameshift variant types, and alternative read coverage ≥10. These variants were located in different genes (*MRPS14*, *UNC13C*, *NXN*, *SLC5A7*, and *ZNF639*) and were all found in metastasis (Table 1). Interestingly, only a missense variant in the *UNC13C* gene was determined in the primary tumor of the patient. According to the ACMG-AMP criteria, all variants were classified as uncertain significance (US).

Mutational signature analysis was carried out using SigProfiler [9]. All possible single nucleotide polymorphisms (SNVs) with their trinucleotide contexts were clustered in 96 possible combinations (SBS96 mode), and then were annotated by COSMIC mutational signatures v.3.3 [13]. All identified somatic variants were related to SBS1 and SBS5 mutational signatures in approximately equal percentage for both samples: 10% vs. 8% of variants for SBS1, and 90% vs. 92% of variants for SBS5 in the primary tumor and metastasis, respectively. 

### 3.3. Copy Number Variation (CNV) Analysis

**Figure 3:**
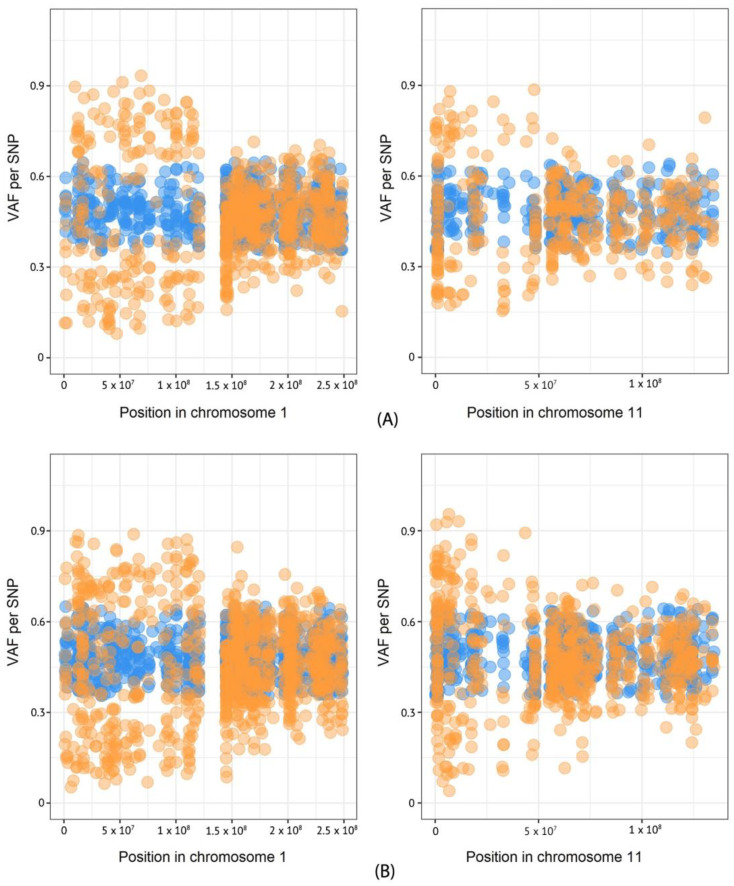
Deletion of chromosome 1p and 11p in primary tumor and metastasis of patient. (**A**) VAF of heterozygous SNPs in primary tumor, (**B**) VAF of heterozygous SNPs in metastasis. Orange dots indicate SNPs in tumor tissue; blue dots mark SNPs in matched norm.

Based on exome sequencing data, we performed the analysis of CNVs in the primary tumor and metastasis of the patient using the beta allele frequency (BAF) method (6). Heterozygous SNPs identified in normal and tumor tissues were plotted on the diagram according to their VAF values, and then the difference between VAF values was estimated using Fisher’s exact test. As a result, deletions of chromosomes 1p and 11p were found in both the primary tumor and metastasis samples (Figure 3).

To verify the loss of heterozygosity of the *SDHB* gene, we carried out the analysis of four fluorescent microsatellite markers (D1S228, D1S507, D1S436, and D1S199) using DNA from the primary tumor, metastasis, and peripheral blood. These markers lie between the 1p36.21 and 1p36.13 regions and cover the *SDHB* locus. Microsatellite analysis was performed with fragment analysis by capillary electrophoresis on a NANOFOR 05 instrument (Syntol, Russia); data were analyzed using the GeneMarker v.1.71 (SoftGenetics LLC, State College, PA, USA). For the patient studied, two markers (D1S228 and D1S507) were homozygous and non-informative, while D1S436 and D1S199 dinucleotide repeats demonstrated a deletion of locus on chromosome 1p in both the primary tumor and metastasis in accordance with results of the BAF analysis (Figure S2).

## 4. Discussion

MEPGLs belong to PPGLs that are often hereditary (at least 30%) [14]. MEPGLs, like all HNPGLs, are associated with germline mutations predominantly in the *SDHx* genes encoding the four subunits of the succinate dehydrogenase (SDH) complex (mitochondrial complex II) [15]. In MEPGLs, germline variants were more frequently found in *SDHC*, followed by *SDHB*, *SDHD*, and *SDHA* genes, while HNPGLs of other localization (carotid and vagal paragangliomas) were characterized by prevalence of mutations in *SDHD* and *SDHB* genes [16,17]. Malignant PPGLs are very rare with an incidence of less than 1 in 1,000,000 [18]. One of the known factors associated with high risk of metastasis is germline mutation in the *SDHB* gene, which is recommended for genetic testing of patients with PPGLs [19]. We identified germline variant p.R230H in the *SDHB* gene in the studied patient, which is in concordance with the reported data about high frequency of *SDHB* mutations in malignant HNPGLs [20]. The Varsome tool classifies *SDHB*: p.R230H as the pathogenic variant basing on the following criteria: (1) PP5, very strong evidence on variant pathogenicity due to its multiple submission in association with disease, (2) PM5, strong evidence resulting from the facts of alternative pathogenic missense changes at the same amino acid residue, (3) PP3, strong evidence supported by deleterious effect on protein predicted by multiple computational tools, (4) PM1, moderate evidence provided from the localization of the variant in the hot-spot region without known benign variations, and (5) PM2, supporting evidence basing on the low population frequency of the variant. In addition to the above, a nematode *Caenorhabditis elegans* model of the *SDHB*: p.R230H mutation (worm’s equivalent mutation *SDHB-1*: p.R244H) showed elevated lactate and pyruvate levels, indicating aberrant glycolysis reminiscent of the Warburg effect’s metabolic reprogramming in cancer [21]. According to the protein molecular structure, arginine in the position 230 forms hydrogen bonds with the carboxyl oxygens of asparatic acid 224. This Asp224–Arg230 salt bridge is a conserved structure, changes in which might impair protein function. The importance of the Asp224–Arg230 contact is confirmed by high frequency of mutations in these positions (Arg230Gly, Arg230Cys, Arg230His, Arg230Leu, and Asp224His) related to hereditary syndromes, including remarkably aggressive recurrent and metastasizing tumors [22,23,24,25]. Moreover, structural models of the *SDHB*: p.R230H mutation revealed a reduction of five inter-residue contacts, as well as loss of contacts at the interface between the distant N-terminal domain of *SDHB* and SDHA that can result in inactivation or aberrant activity of the SDH complex. 

In the studied patient, the BAF method and microsatellite analysis also showed the LOH for chromosome 1p, indicating the absence of the wild-type *SDHB* allele. This finding suggests that *SDHB* acts as a tumor suppressor gene (TSG) and development of MEPGL can occur in accordance to the Knudson’s two-hit hypothesis [26]. Additionally, we determined LOH of the chromosome 11p locus in both the primary tumor and metastasis. According to the Hensen model, the 11p15 region harbors an imprinted TSG that plays an essential role in tumorigenesis of *SDHD*-mutated PPGLs [27]. Loss of the 11p15 region was also found in *VHL*, *SDHAF2*, and *SDHB*-related PPGLs [28]. In sporadic PPGLs, loss of chromosome 11p was more frequent in malignant cases than in benign ones [29]. Thus, loss of 11p seems to act as an important somatic event in tumor development, especially in the course of metastasis. 

Ki-67/MIB1 expression has also been suggested as a potential predictor of malignancy and was included as a parameter in a grading system for adrenal pheochromocytoma and paraganglioma (GAPP) [20,30]. A series of studies were in agreement that Ki-67/MIB-1 labeling index over 2–4% can highlight the malignant potential of PPGLs [31,32,33]. In our study, the Ki-67 antigen staining of the primary tumor revealed proliferation activity of 4% that support the prognostic value of Ki-67 expression for detection of malignant paragangliomas.

Based on whole-exome sequencing data, we found four somatic potentially disease-causing variants associated with metastasis, and a common somatic variant in the primary tumor and metastasis. All variants were first detected in our study and were interpreted as US. However, their pathogenicity was predicted by several in silico algorithms, and all variants had high conservation score and low population frequency. In *MRPS14*, encoding for one of the mitochondrial ribosomal proteins, a small deletion in exon 2 was found. *MRPS14* was not previously reported in association with tumors, but mutations in this gene lead to disruption in mitochondrial translation and multiple respiratory chain deficiency [34]. Possibly, in the presented case of hereditary *SDHB*-linked paraganglioma, a somatic mutation in *MRPS14* may further exacerbate impaired mitochondrial function and contribute to tumor development. Nucleoredoxin, coded by the *NXN* gene, participates in the maintenance of cellular redox homeostasis. It serves as an important regulator for many signaling pathways (WNT/β-catenin, PKB/Akt, and NF-κB) and biological processes, such as cell cycle, growth, proliferation and differentiation, apoptosis, etc. [35]. A wide spectrum of *NXN* interactions leads to its involvement in different human pathologies, including cancer [35]. Herein, we first showed a somatic mutation in the *NXN* gene in metastasis derived from MEPGL, but the associated pathway remains unknown. In metastasis, we also determined somatic missense variants in the *SLC5A7* and *ZNF639* genes, which are members of two large families of proteins, solute carrier (SLC) family of transporters and zinc finger protein (ZNF) family of transcription factors. Both genes were previously noted in association with cancer, and, interestingly, somatic variants in genes belonging to these families have often been found in paraganglioma datasets (NCBI Sequence Read Archive, BioProject PRJNA778918). 

A common somatic variant identified both in the primary tumor and metastasis was revealed in the *UNC13C* gene, encoding for a protein with predicted participation in glutamatergic synaptic transmission and vesicle maturation, as well as regulated exocytosis. Despite not having a fully annotated function, *UNC13C* was earlier identified as a significantly mutated gene in oral squamous cell carcinoma and its decreased expression was correlated with advanced tumor stages and shorter survival of patients [36,37]. B. K. Velmurugan with colleagues showed that *UNC13C* overexpression significantly impaired metastasis and invasive ability of the human tongue squamous carcinoma cell lines SCC-9 and SAS [37]. Our results also indicate that the identified somatic mutation of this gene can contribute to tumor progression.

The higher number of potentially deleterious variants which were identified in metastasis can be explained by higher ML compared with the primary tumor. On the other hand, metastasis with high ML might be enriched by driver mutations that contribute to tumor growth. Notably, mutational load found even in the primary tumor of patients was significantly higher than those reported for non-malignant carotid paragangliomas (1.4/Mb vs. 0.1–0.3/Mb, VAF 15%) [12], but was only one and a half times more than ML in malignant carotid body tumors (0.5/Mb vs. 0.3/Mb, VAF 20%) [38]. Thus, high ML can be a potential indicator for tumor aggressiveness in MEPGL.

Despite the difference in the number of somatic variants in the tumor and metastasis, combinations of mutation types in these samples were similar and generated two mutational signatures, SBS1 and SBS5. Both signatures show clock-like properties. SBS1 mutational signature has been commonly found in many cancers and is proposed to be associated with deamination of 5-methylcytosine to thymine, while SBS5 is characterized by unknown etiology and likely resulted from endogenous background mutational processes [39]. SBS1 and SBS5 mutational signatures are accumulated in almost all tissues throughout life [40]. Possibly, most identified somatic mutations could be generated over the lifespan of patients and were not associated with other specific causal factors in tumor development and progression.

The obtained results are important because they facilitate better understanding of the molecular genetic basis regarding this very rare tumor. The main limitation of the study is that it based on a single medical case. Another drawback is the absence of adjacent normal tissue, which makes it impossible to study the transcriptomic and epigenetic changes associated with the development of a tumor. Additional methods could be used to extend our findings.

## 5. Conclusions

Comprehensive genetic analysis of an extremely rare case of malignant MEPGL revealed the hereditary nature of the disease. The genetic basis for the development and progression of this tumor was a germline *SDHB* p.R230H mutation associated with loss of the gene’s wild-type allele. Moreover, several identified somatic alterations could also contribute to tumor growth; these are loss of chromosome 11p and a somatic variant in the *UNC13C* gene. We also suggest additional potential indicators for ‘malignancy’ of MEPGL (except *SDHB* mutation): high ML and Ki-67 value ≥ 4%. Summarizing, we can advise genetic testing for germline *SDHx* mutations in patients with MEPGLs. Additional tests based on exome sequencing of tumors and normal tissue can help to predict the malignant potential of the disease. However, multi-center studies are required for uncovering disease-associated mechanisms and metastasis markers.

## Figures and Tables

**Table 1:** Somatic potentially deleterious variants identified in tumor and metastasis of the patients.

Gene	Position	Variant Type	Nucleotide Change	Amino Acid Change	Sample	VAF (%)	Deleterious Prediction Tools
*MRPS14*	Chr1: 174987505	Deletion	c.169_204del	p.57_68del	Metastasis	28	-
*UNC13C*	Chr15: 54838976	Missense	c.A5753C	p.E1918A	Primary tumor, metastasis	27, 29	Polyphen2, DANN, SIFT, LRT, MutationTaster, PROVEAN, FATHMM, ClinPred
*NXN*	Chr17: 708424	Missense	c.A884C	p.E295A	Metastasis	28	DANN, LRT, MutationTaster, FATHMM, M-CAP, ClinPred
*SLC5A7*	Chr2: 108627262	Missense	c.T1688C	p.F563S	Metastasis	31	DANN, FATHMM, MetaSVM, MetaLR, M-CAP, ClinPred
*ZNF639*	Chr3: 179051692	Missense	c.G940T	p.D314Y	Metastasis	25	Polyphen2, SIFT, CADD, DANN, LRT, MutationTaster, PROVEAN, ClinPred

## Data Availability

All data generated or analyzed in this study are included in the published article. The exome sequencing data are available in the NCBI BioProject under the accession number PRJNA875276.

## Supplementary Materials

The following supporting information can be downloaded at https://www.mdpi.com/article/10.3390/ijms24098220/s1.
