# Supplementary material for: Comprehensive Genetic Study of Malignant Cervical Paraganglioma
Source: Int J Mol Sci. 2023 May 4;24(9):8220. doi: 10.3390/ijms24098220 (PMC10179044; doi:10.3390/ijms24098220)
Supplement: Supplementary file 1 [file ijms-24-08220-s001.zip › ijms-2354185-supplementary.pdf]

# Supplementary Materials

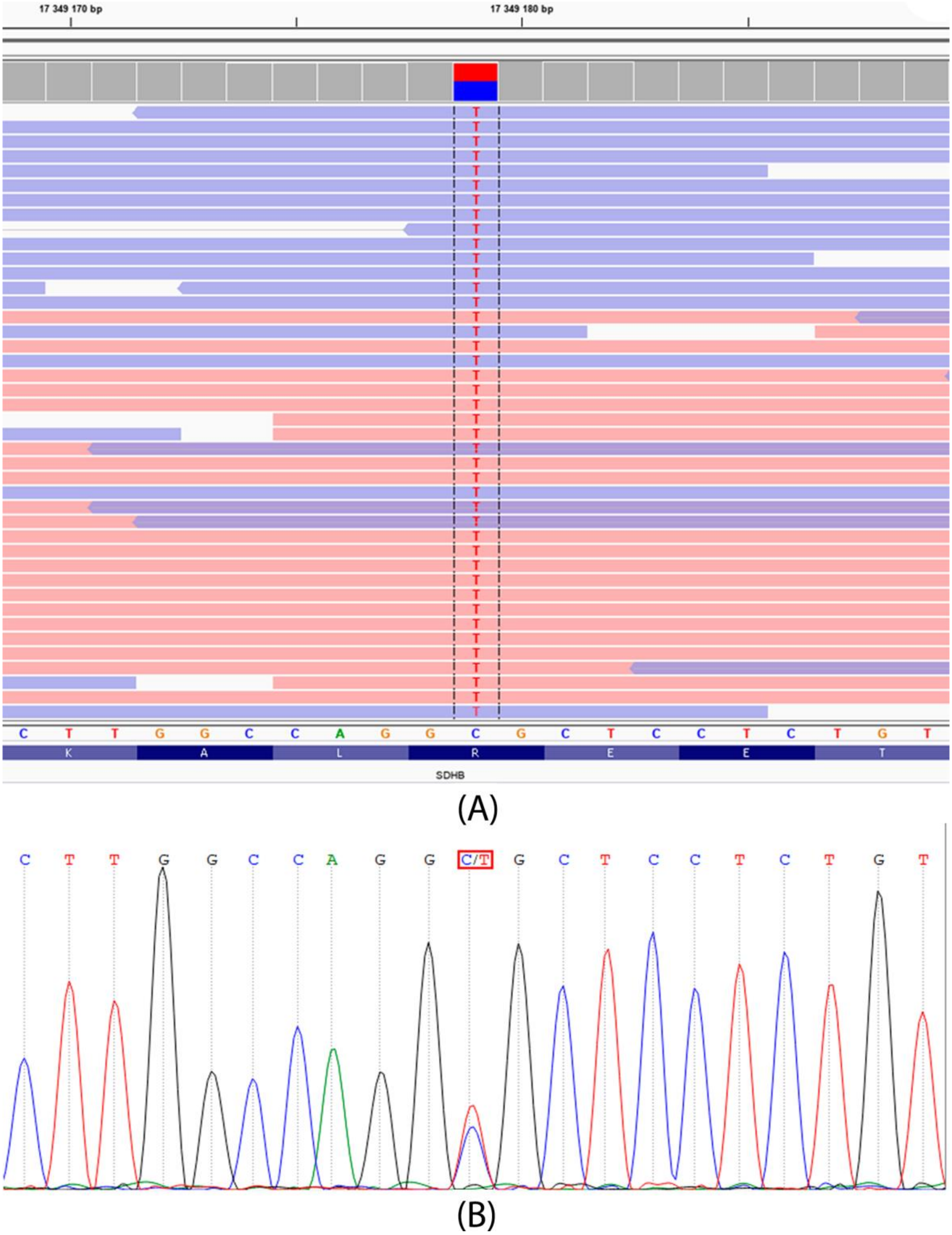


**Figure S1.** Verification of the *SDHB* p.R230H mutation by Sanger sequencing. (**A**) Mapped reads of the exome data from blood sample of the patient in the Integrative Genomics Viewer (IGV) browser (variant nucleotides are marked by red color, forward and reverse reads present as pink and blue horizontal lines, respectively). (**B**) Sanger sequencing chromatogram of the target region in *SDHB* exon 7.

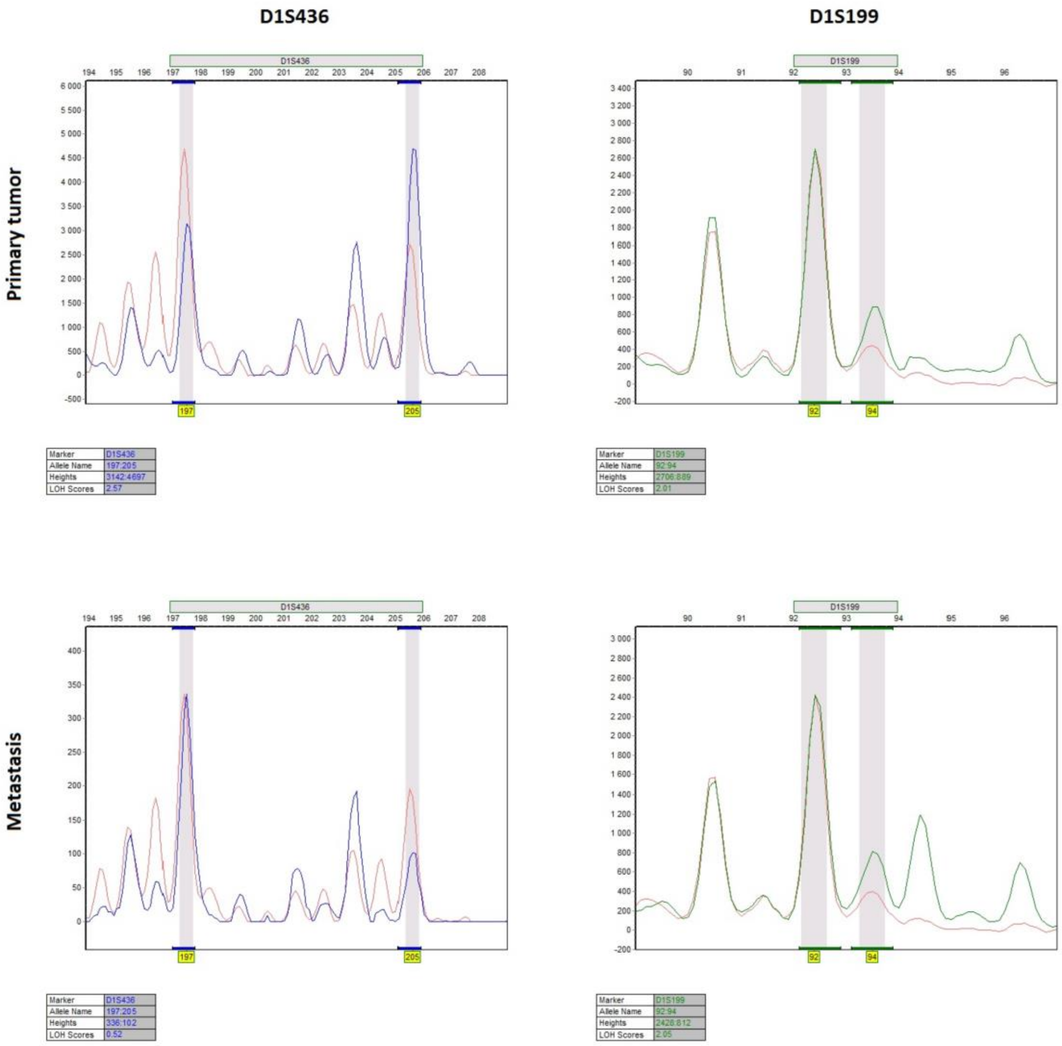


**Figure S2.** Electropherogram traces of LOH at the D1S436 and D1S199 microsatellite markers in primary tumor and metastasis of the patient. The blue and green traces are the tumor DNA overlaid on the red blood DNA trace for visualization of LOH. Y-axis is a relative fluorescent intensity; x-axis indicates length of alleles in nucleotides.
